# Whole-genome sequencing and the clinician: a tale of two cities

**DOI:** 10.1136/jnnp-2013-306264

**Published:** 2014-04-04

**Authors:** A Reghan Foley, Robert D S Pitceathly, Jie He, Jihee Kim, Nathaniel M Pearson, Francesco Muntoni, Michael G Hanna

**Affiliations:** 1 Dubowitz Neuromuscular Centre, MRC Centre for Neuromuscular Diseases, University College London Institute of Child Health and Great Ormond Street Hospital for Children, London, UK; 2 MRC Centre for Neuromuscular Diseases, University College London Institute of Neurology and National Hospital for Neurology and Neurosurgery, London, UK; 3 Knome, Inc., Cambridge, Massachusetts, USA; 4 Foundation Medicine, Inc., Cambridge, Massachusetts, USA; 5 Department of Comparative Biomedical Sciences, Royal Veterinary College, London, UK; 6 Ingenuity Systems/QIAGEN, Redwood City, California, USA

**Keywords:** GENETICS, COLLAGEN, MYOPATHY

## Abstract

**Background:**

Clinicians are faced with unprecedented opportunities to identify the genetic aetiologies of hitherto molecularly uncharacterised conditions via the use of high-throughput sequencing. Access to genomic technology and resultant data is no longer limited to clinicians, geneticists and bioinformaticians, however; ongoing commercialisation gives patients themselves ever greater access to sequencing services. We report an increasingly common medical scenario by describing two neuromuscular patients—a mother and adult son—whose consumer access to whole-genome sequencing affected their diagnostic journey.

**Results:**

Whole-genome sequencing initiated by the patients—to predict their risk of common diseases—revealed that they share several variants potentially relevant to neuromuscular diseases, which initially sidetracked diagnostic efforts. Since eventual clinical reassessment, including muscle imaging, pointed towards Bethlem myopathy, a collagen VI-related myopathy, we pursued Sanger sequencing of *COL6A1*, *COL6A2* and *COL6A3*. This targeted approach revealed a heterozygous causative variant in *COL6A3* (c.6365G>T (p.Gly2122Val)), shared by both individuals, that was *not* flagged by the interpretation of the whole-genome sequencing data.

**Conclusions:**

This report highlights the essential interplay of clinical and genomic expertise in realising the potential of high-throughput sequencing. In an era when patients themselves may bring their own data to the table, definitively identifying clinically significant genomic variants will require close collaboration among clinicians, geneticists and bioinformaticians.

## Introduction

‘We had everything before us, we had nothing before us’

Charles Dickens, *A Tale of Two Cities*

The advent of high-throughput sequencing has ushered in an era of tremendous potential for identifying the molecular causation of simple and complex disorders, rare and common. Advantages of whole-genome sequencing over targeted, exon capture approaches in genetically heterogeneous Mendelian disorders were reported by Lupski *et al* in 2010.^1^ Since then, the identification of new disease genes, as well as causative variants in known genes, has proceeded at a remarkable pace.

The successes of whole-genome sequencing reflect the combined interpretive skills of geneticists, bioinformaticians and clinicians working in close collaboration. Indeed, this essential interplay of complementary genomic and clinical expertise is epitomised by the recent identification of new genes in hitherto molecularly uncharacterised conditions. Importantly, patients’ own independent access to genomic technology may catalyse such interaction but may also steer the diagnostic journey from its conventional path, with varied results. The following case highlights this contemporary medical scenario and serves as an instructive tale.

### Case reports

**Figure 1:**
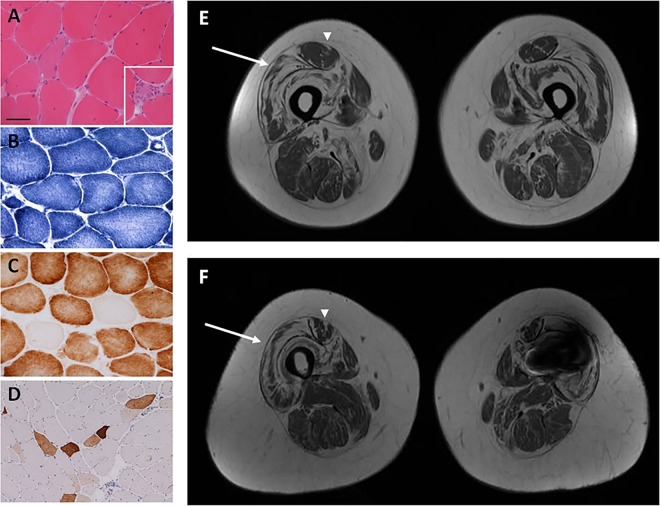
Muscle biopsy performed in the proband reveals variation in fibre size on H&E stain (A) and evidence of necrotic fibres (inset, A). Core-like areas are evident on nicotinamide adenine dinucleotide-tetrazolium reductase (NADH-TR) (B) and cytochrome oxidase (COX) (C) staining. COX-negative fibres are also evident (C). Fibres positive for neonatal myosin likely indicate regenerating fibres (D). Bar in A represents 50 μm in A–C and inset in A, and 100 μm in D. Muscle MRI with axial cuts through mid-thigh region of the proband (E) and his mother (F) reveals abnormal signalling in the central region of the rectus femoris muscle (‘central shadow’ pattern) (arrowheads) and abnormal signalling along the periphery of the vastus lateralis muscle (‘outside-in’ pattern) (arrows). (Signal artefact in left leg of proband's mother (F) due to metallic implant.)

The proband, a 33-year-old male, presented to our (RDSP and MGH) neuromuscular centre in London in 2004. He was never able to run, and he had developed slowly progressive proximal weakness beginning in his teenage years. Examination revealed bilateral elbow contractures, Achilles tendon tightness and proximal muscle weakness (Medical Research Council (MRC)-grade 4/5). Deep tendon reflexes were present. Creatine kinase was 354 IU (reference range: <150 IU). Muscle biopsy revealed evidence of muscle fibre necrosis and regeneration, consistent with an underlying dystrophy (figure 1A–D).^2^

The patient's mother reported a similar history, albeit with a later onset, with proximal muscle weakness noted around age 30 years and progressing to dependence on a cane for walking by age 60 years. Muscle biopsy was reported as dystrophic-appearing but was not available for review.

No other family members were noted to have similar features. The family was counselled that their condition was likely a form of autosomal dominant muscular dystrophy, with Bethlem myopathy and Emery-Dreifuss muscular dystrophy included in the differential diagnosis. Further investigations were recommended; however, the family did not return to our clinic for the ensuing 5 years.

When the family returned in 2010, they brought an electronic data storage device with their annotated whole-genome sequence data, obtained—in order to predict their risk of common diseases—through sequencing contracted through our (JH, NMP) genome interpretation company in Boston. Analysis of the kindred genome data flagged protein-altering non-reference variants in 22 neuromuscular disease-implicated genes that were called in the proband and his mother but not the proband's unaffected father or wife (unrelated), who also underwent whole-genome sequencing. The strongest such candidate, however, was a heterozygous missense variant in *COL6A1* that is modestly common (∼8% allele frequency) in the general population and could not alone plausibly underlie the phenotype.

Two other neuromuscular disease-implicated genes (*SGCA* and *SGCG*) were each called with a heterozygous protein-altering variant shared by the proband and his mother. Variants in these genes have been recessively (or within one gene compound heterozygously) implicated in sarcoglycanopathies, but we knew of no report tracing disease to interaction between heterozygous variants in distinct sarcoglycan genes. Moreover, the clinical phenotype of the patients was not consistent with phenotypes associated with sarcoglycanopathies. As such, the clinical and genome interpretation teams began a close dialogue, to best integrate the initially inconclusive genomic findings with detailed clinical findings, for further diagnostic insight.

In order to further elucidate the patients’ pattern of muscle involvement and decipher the potential relevance of genetic variants of unknown significance, we (ARF, RDSP, FM and MGH) recommended lower extremity muscle MRI be performed, since it can be instrumental in narrowing the differential diagnosis and pointing towards particular genetic aetiologies.^3^ Notably, with this recommendation the patients returned to the original diagnostic pathway. MRI in the proband and his mother revealed abnormal signalling in the central region of the rectus femoris muscle, consistent with a pattern termed ‘central shadow’ and abnormal signalling along the periphery of the vastus lateralis muscle, a pattern described as ‘outside-in’ (figure 1E,F). These muscle MRI findings have been reported in patients with Bethlem myopathy,^4^ ^5^ resulting from pathogenic variants in any of the 3 collagen 6 genes (*COL6A1, COL6A2* and *COL6A3*).^6–8^

Given this muscle MRI pattern strongly evocative of Bethlem myopathy and consistent with our clinical impression, and since causative variant(s) in the collagen 6 genes were not flagged by the interpretation of the whole genome sequencing data, we pursued follow-up long-read (Sanger) sequencing of *COL6A1, COL6A2* and *COL6A3* in a diagnostic laboratory in London. Strikingly, the targeted sequencing and interpretation flagged a heterozygous c.6365G>T (p.Gly2122Val) novel variant in exon 20 of *COL6A3* in the proband and his mother. Such variants, in the triple helical domain of the collagen VI protein, are known to result in aberrant formation of the extracellular matrix protein collagen VI, resulting in a spectrum of collagen VI-related myopathies ranging from Bethlem myopathy to the more severe and allelic condition Ullrich congenital muscular dystrophy.^9^ While Bethlem myopathy typically follows autosomal dominant inheritance, rare autosomal recessive inheritance has been described as well.^10^ ^11^

Skin biopsies performed in the proband and his mother for dermal fibroblast collagen VI immunocytochemistry and flow cytometry studies (performed as described in Kim *et al*^12^) revealed abnormal collagen VI expression (see online supplementary figure S1A,B), thus providing further evidence of collagen VI deficiency. Sequencing of complementary DNA, extracted from skin fibroblasts, confirmed the presence of the *COL6A3* glycine-altering variant called in the proband and his mother's genomic DNA, which did not result in abnormal splicing.

In arriving at a diagnosis of Bethlem myopathy, other clinically similar conditions such as Emery-Dreifuss muscular dystrophy (and its associated cardiac involvement) were excluded. Counselling regarding the natural history of Bethlem myopathy, its autosomal dominant inheritance pattern in this family and the associated risk of recurrence were provided to the proband and his mother in the setting of our (RDSP and MGH) neuromuscular centre. In contrast, when the family received the annotated data of their independently contracted whole-genome sequencing and interpretation, they did not have genetic counselling.

Re-analysis of the whole-genome sequencing data confirmed that the causative *COL6A3* variant was detected, but not interpretively flagged, due to the inadequacy of our (JH, NMP) pipeline (at the time) for predicting functional effects of variants in whole-genome analysis. Specifically, our whole-genome analysis pipeline relied on data published in conjunction with the SIFT (Sorting Intolerant From Tolerant) algorithm to identify, and optionally functionally prioritise, missense variants. At the time, SIFT annotated the *COL6A3* transcript in question on the wrong strand, and our pipeline did not yet correct, or flag, such discrepancies. By contrast, the conventional pipeline used to interpret targeted sequencing of collagen genes successfully flagged the variant in question as functionally relevant/potentially deleterious.

## Discussion

The diagnostic journey outlined here is provocative for several reasons. First of all, whole-genome sequencing independently pursued by our patients did not uncover the causative *COL6A3* variant. Instead, this variant was identified by Sanger sequencing of the collagen 6 genes, motivated by muscle MRI findings, per the conventional diagnostic pathway. While whole-genome reference data have since improved to better flag potentially harmful variants in *COL6A3*, whole-genome sequencing and interpretation will likely remain ‘blind’ to some variants that targeted methods identify more reliably. Such whole-genome shortcomings are inherent to methods of short-read sequencing (patchiness of coverage; and inadequacy of alignment, variant-calling and haplotyping methods) and interpretation (inaccuracy of predictive algorithms; and patchiness of comparative data from healthy controls). Conventional locus-specific databases, such as those available through the Leiden Open Variation Database (LOVD), may contain more reliable data on particular genes than do sources casting a broader net on our genomes. Such locus-specific databases cover only particular genes, and use sundry formats that defy easy integration for whole-genome interpretation, but they will likely remain a cornerstone of interpretation in difficult cases.

Second, this case highlights muscle MRI as a valuable diagnostic tool for Bethlem myopathy, for which it reportedly yielded 96% diagnostic specificity in a study of patients with Bethlem myopathy and other muscular dystrophies presenting with rigid spine.^13^

Third and most importantly, the diagnostic trajectory described here highlights the indispensable role of clinical expertise in the era of genomic medicine. Clinicians, geneticists and bioinformaticians together face the challenge of wading through an immense quantity of data from patient genomes—a challenge compounded if any of them tries to navigate these waters in isolation.

The commercialisation of high-throughput sequencing alters the landscape of information available to patients, yielding vast data but no easy means to definitively interpret it. Notably, clinician-initiated high-throughput sequencing is now widely available, but always requires formal ethics oversight and, if for research, express institutional approval. By comparison, restrictions on consumer-initiated sequencing vary more widely and in some jurisdictions allow detailed genomic data to be directly available to patients and their relatives—data that will often then be presented to a clinician for review, as in this case. Given the rapid decline of the cost of whole-genome sequencing, many clinicians may soon confront the scenario presented here. Indeed, cost is no longer the limiting factor in whole-genome sequencing, which in 2001 cost ∼US$100 000 000 but in 2011 fell below US$10 000 (National Human Genome Research Institute).^14^ Rather, interpretive expertise—genomic and clinical—is emerging as the limiting factor, in temporal and cost terms.

The greatest hurdle to the use of high-throughput sequencing in clinical practice may, in fact, be time. Genomicists, bioinformaticians and clinicians worldwide must work together, carefully and patiently scrutinising immense quantities of genomic data, on a joint quest to identify variants of clinical significance. Without coordinated, dedicated efforts, the course of genomic medicine risks Dickens’ admonition: ‘we had everything before us, we had nothing before us.’^15^

## Supplementary Material

Web figure
