# Supplementary material for: Whole-genome sequencing and the clinician: a tale of two cities
Source: J Neurol Neurosurg Psychiatry. 2014 Apr 4;85(9):1012–5. doi: 10.1136/jnnp-2013-306264 (PMC4145416; doi:10.1136/jnnp-2013-306264)

Supplementary Figure 1 (A) Immunocytochemistry for collagen VI on cultured fibroblasts (non-permeabilised), derived from skin biopsies from a control patient (without neuromuscular disease), the proband and his mother; red = collagen VI; blue = DAPI nuclear stain to demonstrate cell density; measurement bar = 100 µm. The matrix in the proband and his mother is disorganized in appearance. (B) Flow cytometry analysis quantifying collagen VI expression in the cultured fibroblasts (nonpermeabilised) from the control patient (same control studied immunocytochemically, above), the proband and his mother. Collagen VI expression recorded in the proband (32.2%) and his mother (52.6%) is notably decreased when compared to the control (88.6%).

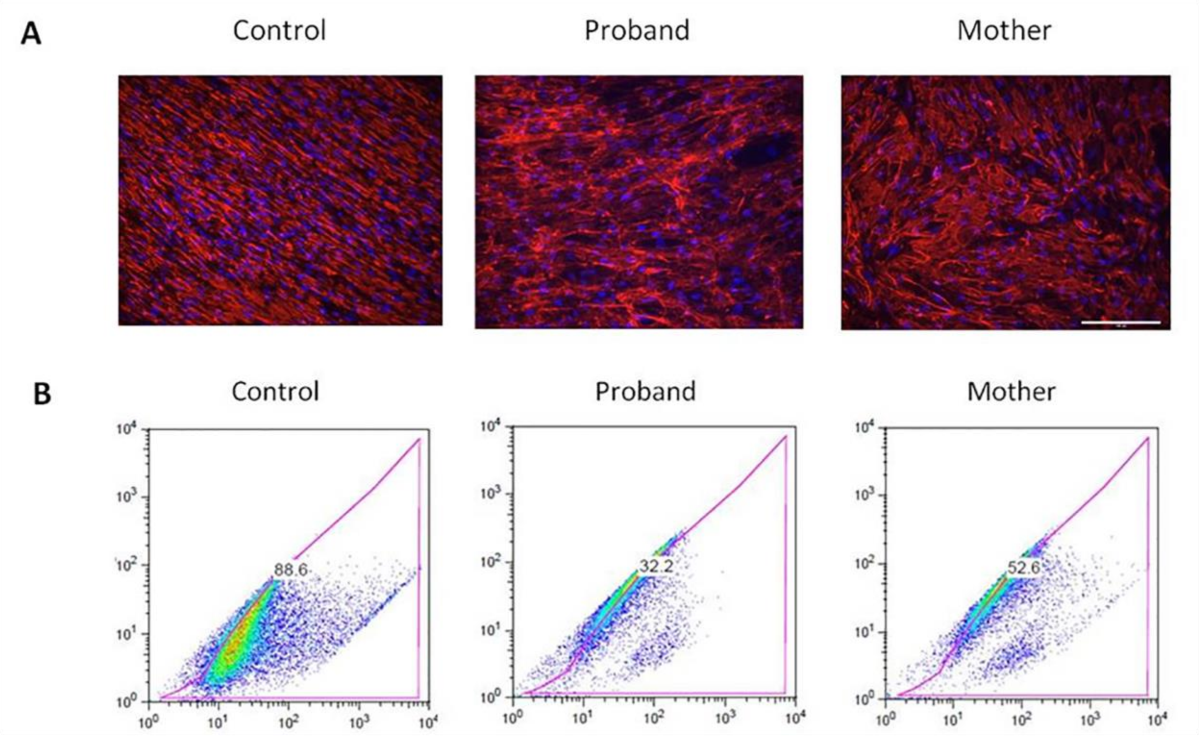

Supplement: Web figure [file jnnp-2013-306264-s1.pdf]
